# Global prevalence, burden and trend in HIV and drug-susceptible tuberculosis co-infection from 1990 to 2019 and prediction to 2040

**DOI:** 10.1016/j.heliyon.2023.e23479

**Published:** 2023-12-10

**Authors:** Longhao Wang, Hengliang Lv, Xueli Zhang, Xin Zhang, Junzhu Bai, Shumeng You, Xuan Li, Yong Wang, Jingli Du, Yue Su, Weilin Huang, Yingzhong Dai, Wenyi Zhang, Yuanyong Xu

**Affiliations:** a Department of Health Statistics, Faculty of Military Preventive Medicine, Army Medical University, Chongqing, China; b Chinese PLA Center for Disease Control and Prevention, Beijing, China; c Department of Epidemiology, School of Public Health, China Medical University, Shenyang, China; d Changchun University of Chinese Medicine, Changchun, China; e Department of Epidemiology and Biostatistics, School of Public Health, Anhui Medical University, Hefei, China; f Tuberculosis Prevention and Control Key Laboratory/Beijing Key Laboratory of New Techniques of Tuberculosis Diagnosis and Treatment, Senior Department of Tuberculosis, The 8th Medical Center of PLA General Hospital, Beijing, China; g College of Basic Medicine, Army Medical University, Chongqing, China

**Keywords:** HIV, Acquired immunodeficiency syndrome, Drug-susceptible tuberculosis, Prevalence, Burden, Joinpoint regression, Prediction

## Abstract

**Objectives:**

The purpose of this study is to describe the current situation and forecast the trends of co-infection between the human immunodeficiency virus (HIV) and drug-susceptible tuberculosis (DS-TB) in different countries, across various age groups and genders.

**Methods:**

We obtained data on the number of cases, age-standardized incidence rate, age-standardized prevalence rate, age-standardized rate of disability-adjusted life years (DALYs), and age-standardized death rate from the Global Burden of Disease (GBD) 2019 database. These data were used to describe the distribution and burden of co-infection between the human immunodeficiency virus (HIV) and DS-TB in different regions, genders, and age groups. We employed joinpoint regression analysis to analyze the temporal trends from 1990 to 2019. Additionally, an age-period-cohort model was established to forecast the future trends of co-infection up to 2040.

**Results:**

The prevalence and burden of co-infection varied across different age groups and genders. The territories with the higher disease burden were distributed in some Asian and African countries. In terms of temporal trends, the age-standardized incidence rate and age-standardized prevalence rate of HIV and DS-TB co-infection exhibited an overall increasing trend from 1990 to 2019, and the prediction indicated a slow downward trend from 2019 to 2040.

**Conclusions:**

The co-infection of HIV and DS-TB posed a grave threat to public health and economic development. What’s more, there existed a significant disparity between the actual state of co-infection and the desired goals for prevention and control.

## 1 Introduction

Acquired immune deficiency syndrome (AIDS) and tuberculosis (TB) are two primary infectious causes of disease burden globally, with TB being the leading cause of death among individuals infected with human immunodeficiency virus (HIV) [1]. The impact of AIDS and TB on morbidity and mortality is significant, and they are increasingly recognized as major global public health concerns [2,3]. About 10.6 million people were estimated to be infected with TB [3], while 1.5 million people were living with HIV worldwide by 2021 [2]. After a long incubation period, AIDS is characterized by low levels of CD4^+^T lymphocytes and a low functioning immune system, allowing infection by various opportunistic pathogens. HIV infection increases susceptibility to *Mycobacterium tuberculosis* (MTB) and promotes the activation of this opportunistic pathogen in the body, while infection with MTB also promotes the progression of AIDS [4]. The complex interaction between these two pathogens contributes significantly to the high disease burden experienced [5]. Unfortunately, there is no known cure for AIDS and TB, which results in substantial damage to the human body, leading to high rates of morbidity and mortality.

The outcomes of HIV and TB co-infected patients have significantly improved due to the implementation of various public health interventions, including antiretroviral therapy (ART) and TB prophylaxis therapy [6]. However, Africa continues to have the highest incidence and number of HIV and TB co-infections, and the situation in Eastern Europe is also concerning due to the high incidence and frequency of drug resistance. Additionally, co-infection tends to be more prevalent among vulnerable populations, such as immigrants [7]. Medical workers in many countries have reported on the epidemiology and disease characteristics of HIV and TB co-infection in recent decades. However, these studies have been limited by small sample sizes frequently. Therefore, conducting a study based on authoritative global data is significant and necessary.

The Global Burden of Disease (GBD) 2019 has recently emerged as a valuable tool for conducting epidemiological investigations, and numerous high-quality papers based on the GBD study have been published in The Lancet [8,9]. In our study, we utilized the GBD database to extract and analyze the burden of HIV and drug-susceptible tuberculosis (DS-TB) co-infection over the past 30 years (1990–2019), examining its relationship to regions, ages, genders and contribute to this expanding field of study by analyzing time series data and predicting future trends in disease burden, which can assist healthcare workers and health departments in making targeted decisions to address the challenges posed by HIV and DS-TB co-infection.

## 2 Methods

### 2.1 Data source

The GBD 2019 is a publicly available database that provides comprehensive information on the burden and prevalence of 369 injuries and diseases across 204 countries and territories from 1990 to 2019. Previous reports have described the details of GBD 2019 extensively [9], and there have been prior studies examining articles relevant to AIDS, tuberculosis, and their co-infection [[10], [11], [12]]. For our research, we extracted the entire dataset on HIV and drug-susceptible tuberculosis (DS-TB) co-infection from the GBD 2019. We utilized the Global Health Data Exchange query tool (http://ghdx.healthdata.org/gbd-results-tool), a web-based tool, to access the data. Specifically, we collected information on the incidence, prevalence, death, Disability Adjusted Life Year (DALY), and age-standardized rate associated with HIV and DS-TB co-infection.

### 2.2 Disease description

For this study, we obtained the incidence rate, prevalence, DALYs rate, and death rate of HIV and DS-TB co-infection. These measures were used to analyze the distribution of different genders and age groups from 1990 to 2019. In order to assess the burden of diseases in 2019, we utilized the data of incidence, prevalence, death, DALYs, as well as the age-standardized incidence rate, age-standardized prevalence rate, age-standardized rate of DALYs, and age-standardized death rate. Additionally, we examined the annual rate of change of HIV and DS-TB co-infection in different countries and regions to understand the trends over time.

### 2.3 Temporal trend analysis

To analyze the temporal trend from 1990 to 2019 at the global level, we employed Joinpoint regression analysis. This statistical method helps identify significant joinpoints, which are points where the trend changes significantly. The approach involves dividing the trend into multiple phases and calculating the annual percentage change and its 95% confidence interval (*CI*) for each phase. The average annual percentage change is then used to summarize the overall change trends from 1990 to 2019. A rising trend is indicated if the annual percentage change value is greater than zero, while a decreasing trend is indicated by a negative annual percentage change value. To determine whether a trend is statistically significant, we consider the probability (*P*) value. If the *P* value is lower than 0.05, we conclude that the trend is significant. Conversely, if the *P* value is greater than 0.05, the trend is considered stable.

### 2.4 Predictions of disease burden

We utilized the GBD data from 1990 to 2019, along with population projections and demographic standardization data, to forecast trends in age-standardized incidence rate, age-standardized prevalence rate, age-standardized rate of DALYs, and age-standardized death rate. To account for the impact of age structure and population dynamics on morbidity, prevalence, DALYs, and mortality, we employed an Age-period-cohort model [13], which is based on generalized linear models, including the power model and the Poisson log-link model. To calculate the predicted age-standardized incidence rate, we converted the incidence of each age group in the 5-year group to the incidence of each age group in each year using a linear relationship. The same approach was used to obtain the predicted age-standardized prevalence rate, age-standardized rate of DALYs, and age-standardized death rate [14]. The R programming language, specifically version 4.2.1, was used to implement the model through the nordpred package. It has been demonstrated that this model performs well in predicting future cancer incidence trends in other study [15].

### 2.5 Statistical analysis

All descriptive studies, visualizations, and predictive analysis were conducted using ArcMap (version 10.8) and R software (version 4.2.1). In addition, we employed Joinpoint regression software (version 4.9.1.0) for temporal trend analysis. A significance level of 0.05 was used, indicating that probability values below this threshold were considered statistically significant.

## 3 Results

### 3.1 The population distribution of HIV and DS-TB co-infection

Fig. 1 illustrated the variations in incidence rate, prevalence rate, DALYs rate, and death rate across different genders and age groups from 1990 to 2019. The findings clearly demonstrated that the index for women was consistently higher than that for men. All four indicators exhibited an initial increase, reaching a peak in 2003–2004, followed by a subsequent decrease towards a lower level. Another meaningful finding was that the age played a significant role in the results, the incidence rate and prevalence rate were consistent in males and females, which were higher between the ages of 20–40, whereas the DALYs rate and death rate indicators were predominant in the age group of under 5 years in both genders.

**Fig. 1:**
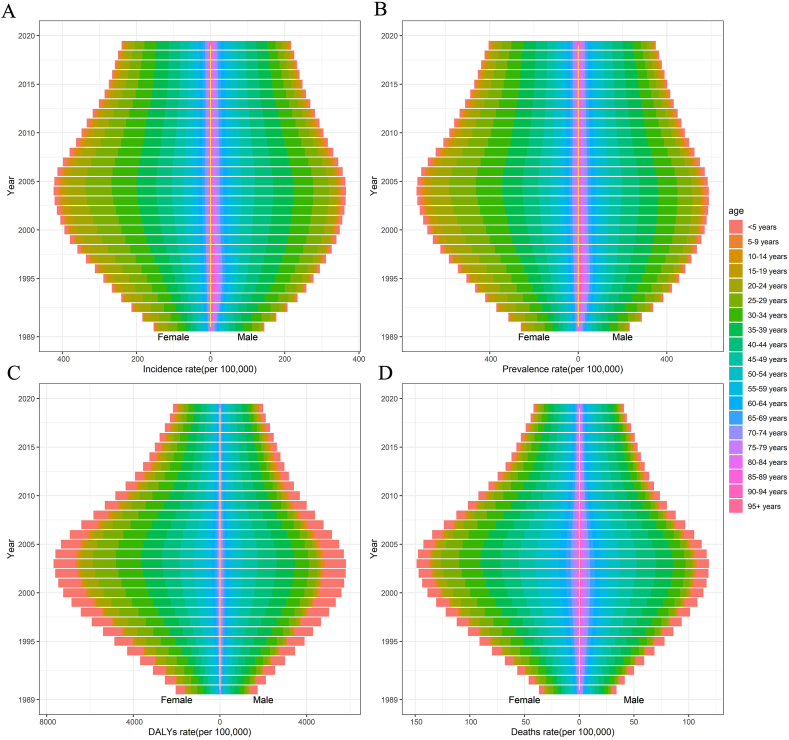
The population distribution of incidence rate (A), prevalence (B), DALYs rate (C) and death rate (D) of HIV and DS-TB co-infection at the global from 1990 to 2019. Note: DALYs: Disability-adjusted life years; HIV: immunodeficiency virus; DS-TB: drug-susceptible tuberculosis.

### 3.2 The region distribution of HIV and DS-TB co-infection

**Fig. 2:**
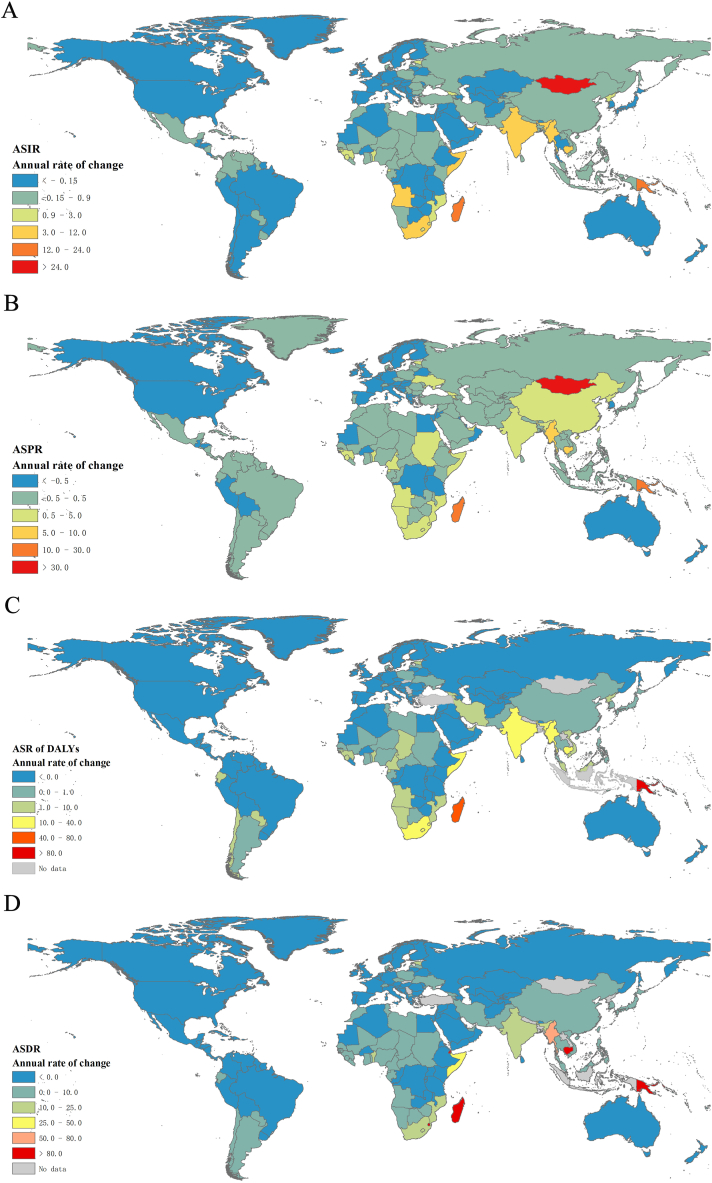
The annal change of rate of ASIR (A), ASPR (B), ASR of DALYs (C) and ASDR (D) in different countries and regions from 1990 to 2019. Note: ASIR: age-standardized rate; ASPR: age-standardized prevalence rate; ASR of DALYs: age-standardized of Disability-adjusted life years rate; ASDR: age-standardized death rate.

In terms of disease burden, Africa had a more severe burden compared to other regions in 2019. South Africa had the highest number of patients (315,902; 95% UI: 275,073 to 359,917), number of DALYs (1,915,233; 95% UI: 1,177,166 to 2,744,386), and number of deaths (37,333; 95% UI: 21,639 to 55,053) (Fig. S1). Additionally, the annual rate of changes was analyzed from 1990 to 2019. Fig. 2 illustrated that Mongolia had the highest age-standardized incidence rate and age-standardized prevalence rate, followed by Madagascar (age-standardized incidence rate = 23.59, 95% UI: 20.65 to 26.88; age-standardized prevalence rate = 22.36, 95% UI: 19.58 to 25.58) and Papua New Guinea (age-standardized incidence rate = 22.35, 95% UI: 20.53 to 24.10; age-standardized prevalence rate = 28.55, 95% UI: 26.07 to 30.72). In terms of age-standardized rate of DALYs and age-standardized death rate, Papua New Guinea had the highest age-standardized rate for DALYs (105.83, 95% UI: 35.53 to 369.36) and Madagascar followed closely (77.38, 95% UI: 29.93 to 261.48). Djibouti also had a relatively high age-standardized rate for DALYs (61.31, 95% UI: 20.51 to 194.74) and age-standardized death rate (112.25, 95% UI: 28.51 to 995.85).

### 3.3 The temporal trends of HIV and DS-TB co-infection from 1990 to 2019

**Fig. 3:**
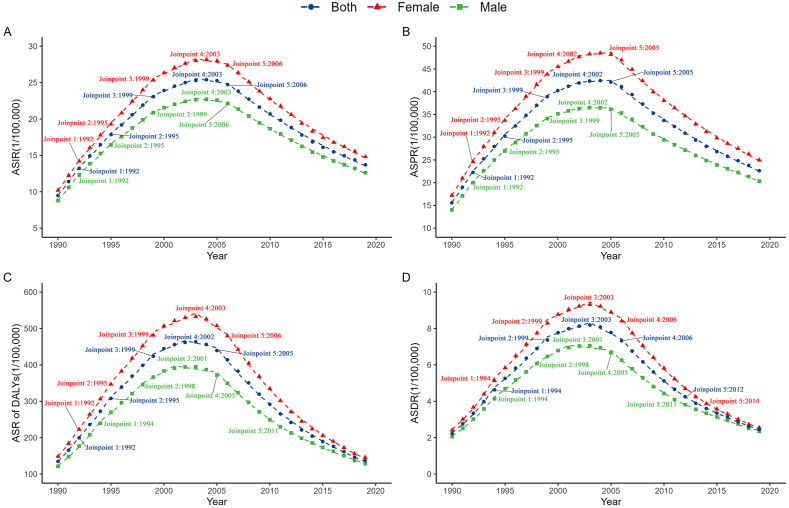
Joinpoint regression analysis of ASIR (A), ASPR (B), ASR of DALYs (C) and ASDR (D) of HIV and DS-TB co-infection at the global from 1990 to 2019. Note: ASIR: age-standardized rate; ASPR: age-standardized prevalence rate; ASR of DALYs: age-standardized of Disability-adjusted life years rate; ASDR: age-standardized death rate.

Average annual percentage change by Joinpoint regression analysis was presented in Table 1 and Fig. 3. The age-standardized incidence rate of HIV and DS-TB co-infection exhibited an overall increasing trend from 1990 to 2019 (average annual percentage change = 1.2, 95% *CI*: 0.8 to 1.7, *P* < 0.001). However, there was a tendency for an initial increase followed by a subsequent decrease. The turning point for age-standardized incidence rate values occurred in 2003 (Fig. 3A). Similarly, the age-standardized prevalence rate of HIV and DS-TB co-infection showed a similar pattern to age-standardized incidence rate, with an overall increasing level (average annual percentage change = 1.3, 95% *CI*: 0.8 to 1.7, *P* < 0.001) and an upward, followed by a downward trend (Fig. 3B). Regarding the age-standardized rate of DALYs (Fig. 3C) and age-standardized death rate (Fig. 3D), only the age-standardized death rate for males showed statistical significance. It displayed an increase before 2001 and then consistently decreased. Furthermore, the age-standardized death rate level in 2019 was slightly higher than in 1990 (average annual percentage change = 0.4, 95% *CI*: 0.0 to 0.8, *P* = 0.029).

**Table 1:** The temporal trends of HIV and DS-TB co-infection at the global from 1990 to 2019.

Measure	Sex	AAPC	95%CI			P
			Lower	Upper		
ASIR	Male	1.2	0.9	1.6	<0.001	
	Female	1.2	0.7	1.8	<0.001	
	Both	1.2	0.8	1.7	<0.001	
					
ASPR	Male	1.3	0.9	1.6	<0.001	
	Female	1.3	0.8	1.7	<0.001	
	Both	1.3	0.8	1.7	<0.001	
					
ASR of DALYs	Male	0.2	−0.2	0.5	0.417	
	Female	−0.1	−0.8	0.5	0.662	
	Both	0	−0.7	0.7	0.999	
					
ASDR	Male	0.4	0	0.8	0.029	
	Female	0.1	−0.3	0.5	0.623	
	Both	0.3	−0.1	0.6	0.159

### 3.4 The prediction of HIV and DS-TB co-infection to 2040

**Fig. 4:**
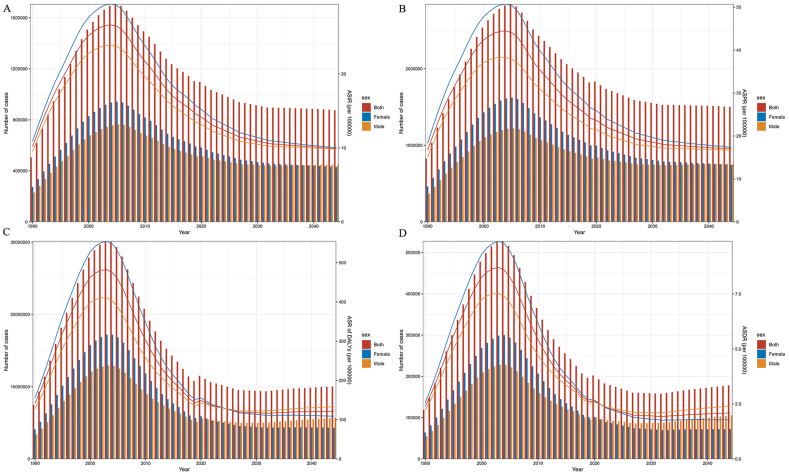
The prediction of incidence rate/number (A), prevalence/number of case (B), DALYs rate/number (C) and death rate/number (D) of HIV and DS-TB co-infection at the global to 2040. Note: DALYs: Disability-adjusted life years; HIV: immunodeficiency virus; DS-TB: drug-susceptible tuberculosis.

According to our projections, the age-standardized incidence rate, age-standardized prevalence rate, age-standardized rate of DALYs, and age-standardized death rate were expected to continue their downward trend and remain stable from 2020 to 2040 (Fig. 4). Furthermore, an interesting finding from 1990 onwards is the significant impact of gender on the results. The age-standardized incidence rate (Fig. 4A) and age-standardized prevalence rate (Fig. 4B) consistently showed higher values in women compared to men. However, there are two notable exceptions: the age-standardized rate of DALYs achieved higher values for men in 2024, and the age-standardized death rate surpassed women in 2021 (Fig. 4C and D).

## 4 Discussions

The prevalence and burden of HIV and DS-TB co-infection remained alarming until 2019, suggesting that the existing prevention and control strategies have not fully met the requirements [2,10]. In this study, we performed an analysis of the prevalence and burden in relation to sex, age, and region. Additionally, we explored the temporal trends of the prevalence and burden of disease and utilized a specific model to make objective forecasts, which revealed a pattern of initial increase followed by a subsequent decrease. This suggested that despite the worsening disease epidemics in previous years, factors such as expanded health care coverage, advancements in prevention and treatment, and improved public health environments may have contributed to the observed declines [9,15].

The study revealed that regional differences significantly impact the incidence of HIV and DS-TB co-infection. Among various factors, income and wealth play a critical role in an individual's health [16,17]. While the global trend has shown a reversal since 2003, many countries still faced high levels of incidence in 2019. The countries experiencing the highest increase of the disease’s burden were primarily concentrated on Mongolia, Madagascar, Papua New Guinea, and Djibouti, and South Asia, East Asia, and some African countries were followed. These countries are generally classified as nations with limited social resources and lower levels of economic development. However, The burden of HIV and DS-TB co-infection leads to increased government spending, prevention and control strategies are not fully implemented in developing countries, further exacerbating the disease burden and creating a vicious cycle [18]. Another study has found that successful social protection and poverty eradication programs can reduce morbidity and mortality rates [19], which suggested that health and population ministries to collaborate with finance ministries and non-governmental organizations to develop protection systems for impoverished families [20]. In this study, we acknowledged that there might be underestimations even absences due to incomplete epidemiological coverage, limited diagnostic capacity, weak disease screening systems, and restricted access to healthcare in some countries [21]. But more meaningful would be the inclusion of comprehensive geographic coverage and analysis of the changing trends in disease burden in 204 countries worldwide over a wide time span, which were not addressed in relevant studies [9,16].

In our study, age and gender were found to be significant factors. We observed that the incidence rate, prevalence, DALYs rate, and death rate were higher in women compared to men, and this difference could primarily be attributed to higher viral loads [22,23], stronger immune activation [24], and changes in microbiota [25] among women. Regarding the incidence rate and prevalence of HIV and DS-TB co-infection, we found that the main affected population groups were concentrated among the young. Factors such as smoking, drug use, and sexual activity were identified as the main causes of exposure to risk [26], and poor environments, malnutrition, and lack of protection were even more severe in low-income countries [27]. Young children comprise a significant portion of the DALYs rate and death rate, which could be attributed to the fact that co-infection has a particularly negative impact on the quality of life and survival of infants who have underdeveloped immune systems. On the other hand, in the elderly population, HIV accelerated cellular and molecular mechanisms of aging, such as mitochondrial abnormalities, epigenetic changes, and DNA damage, leading to increased mortality [28,29]. Notably, the study offered a more detailed analysis of population information compared to other studies [30,31]. Specifically, we have compared the burden of HIV and DS-TB co-infection in different age groups and sexes from 1990 to 2019.

Not only does the article describe the trend of co-infection from 1990 to 2019, but also predicts the year to 2040 which can provide limited reference for the health department and help them make timely adjustments to future health policies. We observed that all indicators reached their peak in 2003 and 2004, followed by a gradual decline that can be largely attributed to the global implementation of highly active antiretroviral therapy (HAART) and the implementation of the WHO policy paper on TB and HIV co-infection [9,15]. In the forecast section, a slight upward trend in the age-standardized rate of DALYs and age-standardized death rate was found around 2020, which aligns with the “Global tuberculosis report 2022” [3]. It is worth noting that the COVID-19 pandemic may have had a detrimental effect on the accessibility and affordability of disease diagnosis and treatment [32]. Consequently, there may be a reduction in the number of diagnoses and reports, leading to incomplete epidemiological data. Additionally, the rehabilitation and treatment of co-infections may be negatively impacted by COVID-19, thereby increasing the disease burden to some extent. According to the strategic target set by the WHO, the number of people with HIV dying from TB, hepatitis B, and hepatitis C should be controlled to 55,000 by 2030 [33], however, the target is exceeded by HIV-TB co-infection alone according to our projections. Our prediction found that the co-infection situation may decrease in the future, but the decline was not obvious, and the control goal of the two diseases and co-infection would not be achieved according to this trend [34,35]. This situation should be recognized and addressed by the health department, which should take appropriate actions to meet this challenge, when TB patients are found, HIV screening is also performed at the same time, for example.

Our study has a few limitations that should be acknowledged. Firstly, epidemiological studies often lack precise exposure information, which makes it difficult to quantify risk accurately. Secondly, the GBD results are derived from a combination of system dynamics models and statistical models, but without real observational data, the estimates may not be entirely accurate. Thirdly, the lack of effective health surveillance systems in low-income countries can result in an underestimation of disease burden. Lastly, the absence of data for certain countries creates gaps in regional descriptions and may have an impact on other results.

## 5 Conclusions

HIV/AIDS, TB, and their co-infection continue to pose significant threats to public health and economic development. According to our study, the disease burden of co-infection has shown a downward trend in recent years, which is expected to continue in the future, as predicted by our established model, however, it is important to note that the current downward trend still falls short of the expected target. The burden of co-infection remains a concerning situation, and efforts to prevent and control co-infection face significant challenges.

## Data availability statement

The datas used were publicly for this study. The website of the data is: https://vizhub.healthdata.org/gbd-results/.

## Ethics declarations

Informed consent was not required for this study because the data used in this study came from public databases, and did not involve animal and human experiments, as well as other data related to human privacy.

## Funding

This study was funded by grants from the 10.13039/501100001809National Natural Science Foundation of China (12031010), the Special Grant for the Prevention and Control of Infectious Diseases (2018ZX10713003). The funders had no role in study design, data collection and analysis, the decision to publish, or the preparation of the manuscript.

## CRediT authorship contribution statement

**Longhao Wang:** Writing - original draft, Software, Resources. **Hengliang Lv:** Writing - review & editing, Writing - original draft. **Xueli Zhang:** Writing - original draft. **Wenyi Zhang:** Writing - review & editing, Validation, Supervision. **Xin Zhang:** Data curation. **Junzhu Bai:** Software. **Shumeng You:** Formal analysis. **Xuan Li:** Visualization. **Yong Wang:** Project administration. **Jingli Du:** Data curation. **Yue Su:** Methodology. **Weilin Huang:** Conceptualization. **Yingzhong Dai:** Data curation. **Yuanyong Xu:** Validation, Funding acquisition, Conceptualization.

## Declaration of competing interest

The authors declare that they have no known competing financial interests or personal relationships that could have appeared to influence the work reported in this paper.

## Figures and Tables

**figs1:**
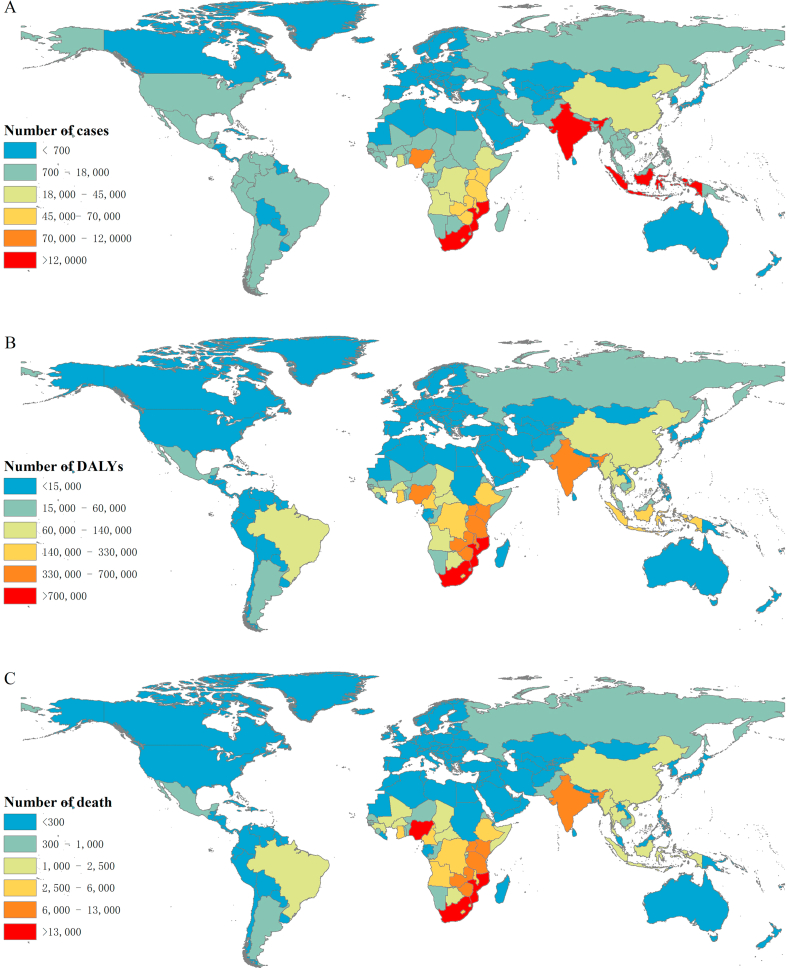

